# Flow Virometry in Wastewater Monitoring: Comparison of Virus-like Particles to Coliphage, Pepper Mild Mottle Virus, CrAssphage, and Tomato Brown Rugose Fruit Virus

**DOI:** 10.3390/v17040575

**Published:** 2025-04-16

**Authors:** Melis M. Johnson, C. Winston Bess, Rachel Olson, Heather N. Bischel

**Affiliations:** Department of Civil and Environmental Engineering, University of California Davis, Davis, CA 95616, USA; meljohnson@ucdavis.edu (M.M.J.);

**Keywords:** flow cytometry, wastewater treatment, viral surrogates, PMMoV, CrAss, ToBRFV, UV disinfection, coliphage, process monitoring

## Abstract

Flow virometry (FVM) offers a promising approach for monitoring viruses and virus-like particles (VLPs) in environmental samples. This study compares levels of non-specific VLPs across a wastewater treatment plant (WWTP) with levels of somatic coliphage, (F+) specific coliphage, Pepper Mild Mottle Virus (PMMoV), CrAssphage (CrAss), and Tomato Brown Rugose Fruit Virus (ToBRFV). All targets were quantified in influent, secondary-treated effluent, and tertiary-treated effluent at the University of California, Davis Wastewater Treatment Plant (UCDWWTP) over 11 weeks. We established an FVM-gating boundary for VLPs using bacteriophages T4 and ϕ6 as well as four phages isolated from wastewater. We then utilize T4 alongside three submicron beads as quality controls in the FVM assay. Coliphage was measured by standard plaque assays, and genome copies of PMMoV, CrAss, and ToBRFV were measured by digital droplet (dd)PCR. FVM results for wastewater revealed distinct microbial profiles at each treatment stage. However, correlations between VLPs and targeted viruses were poor. Trends for virus inactivation and removal, observed for targeted viruses during wastewater treatment, were consistent with expectations. Conversely, VLP counts were elevated in the WWTP effluent relative to the influent. Additional sampling revealed a decrease in VLP counts during the filtration treatment step following secondary treatment but a substantial increase in VLPs following ultraviolet disinfection. Defining application boundaries remain crucial to ensuring meaningful data interpretation as flow cytometry and virometry take on greater significance in water quality monitoring.

## 1. Introduction

Flow cytometry (FCM) was first introduced in the mid-20th century as a technique to count cells by aligning them in sheath fluid and measuring how they scattered light [1]. While initially designed to measure cells and bacteria, advancements in optical systems, fluorescence detection, laser technologies, and the instrument throughput have transformed FCM into a versatile tool that facilitates the rapid characterization and enumeration of particles varying in size, complexity, and biochemical composition [2]. While FCM is most widely recognized for its applications in human medical and biological cell analysis, particularly in immunology, it has also proven valuable in a range of other fields including food and beverage production, microbiology, virology, small particle detection, and microbial monitoring in water and wastewater treatment [3,4]. Hercher et al. (1978) first reported the detection of individual viruses using FCM, noting differences in FCM profile characteristics between viruses and submicron beads (as small as 0.091 microns) [5]. Since then, numerous studies have demonstrated the capability of flow cytometry to detect viruses of varying biochemical structures and environmental origins, redefining FCM applications to viruses as flow virometry (FVM) [4]. FVM studies have detected viral targets isolated from diverse environments, including cell lysates, soils, freshwater systems, marine sediment, and wastewater treatment facilities [6,7,8,9,10,11].

FCM has proven valuable for online, near real-time monitoring of bacterial water quality and microbial treatment efficiency in wastewater treatment plants (WWTPs) [12]. Wastewater influent originates from various sources including households, industries, and surface runoff which contains human, animal, and food wastes that harbor a wide variety of viral hosts [1]. Proper treatment, including virus removal or inactivation, is essential to mitigate risks to human health and the environment downstream of treated effluent. Virus monitoring in wastewater is thus an important tool for evaluating the prevalence of pathogens of interest within a sewershed and identifying potential outbreaks, evaluating process treatment performance, informing regulations related to treatment operations and public health, and assessing potential downstream risks of human exposure to waterborne viruses [13,14]. FCM has been used to monitor bacterial and protozoan levels across multiple stages of wastewater treatment as an alternative to conventional microbial water quality indicators, such as heterotrophic plate counts (HPC) [15,16]. The potential for enumeration of viruses in wastewater via FVM has also been demonstrated at specific treatment stages [10]. Unfortunately, reports of non-specific virus-like particles (VLPs) determined by FVM often lack appropriate quality controls that enable method validation, reproducibility, and translation of FVM methods across laboratories. We previously outlined protocols for VLP enumeration in environmental waters that include bacteriophage T4 as an intact virus positive control and sub-micron-sized fluorescent beads as internal controls [11]. Studies are needed to compare validated VLP counts across multiple stages within a WWTP against results using other direct methods for virus monitoring.

Viruses can be enumerated in wastewater directly by a range of techniques, with cell-culture assays and molecular methods such as genetic sequencing and polymerase chain reaction (PCR) techniques most common in wastewater monitoring [14]. Cell culture methods expose host cells to samples containing target viruses to assess virus infectivity and are regarded as the gold standard for the detection of infectious viruses [17]. *Standard Methods for the Examination of Water and Wastewater, 24th edition* references two methods for enumerating infectious viruses through culture-based plaque assays: standard method #9224—detection of coliphages and standard method #9510—detection of enteric viruses [18]. Culture methods are relatively time-consuming (typically requiring a day or longer to obtain results), tend to lack sensitivity and precision in complex environmental matrices like wastewater, and may underestimate health risks due to their inability to enumerate viable but not culturable viruses [19,20]. Molecular techniques for quantifying viral nucleic acids using PCR are highly accurate and sensitive, but they involve greater startup costs compared to coliphage culture methods. Widely adopted and standardized PCR assays for the detection of viral pathogens in wastewater monitoring also do not differentiate between infectious and non-infectious viruses [14]. PCR-based detection of non-human viruses, including Pepper Mild Mottle Virus (PMMoV) and CrAssphage (CrAss), has been increasingly applied to assess viral removal efficiencies due to their high titer and consistent prevalence in wastewater [21].

In California, WWTPs that recycle water are required to demonstrate the effective removal of viruses through process-specific studies or challenge tests before beginning operation [13]. For ongoing process control, facilities have the option of reporting surrogate parameters (such as TDS and electrical conductivity) of process performance rather than monitoring virus removal directly. Monitoring surrogate parameters is often conducted in lieu of direct virus quantification due to cost and time efficiencies [13,20]. FVM offers a biologically relevant alternative to other high-throughput surrogate parameters used for monitoring virus removal during wastewater treatment. FVM detects viruses or VLPs by using fluorescence labeling to differentiate viral signals from other particles [2]. Huang et al. (2019) applied SYBR Gold (a membrane and capsid penetrating stain that binds with DNA and RNA) to non-specifically stain wastewater samples and used the fluorescence intensity of the stained particles to differentiate bacteria and suspected VLPs at different stages of treatment for three WWTPs [10]. FVM methods for specific detection of viruses include protein labeling with antibodies and fluorescent tags, genomic labeling using nucleic acid stains or in situ hybridization, and membrane staining using lipid and cytosolic dyes [22,23]. Previous studies suggest that virus counts analyzed by FVM are generally one to two orders of magnitude higher than their enumeration as plaque-forming units (PFU) by culture-based assays, but this can vary by virus type and FVM method [11,24]. Challenges for application of FVM in water quality monitoring include a lack of standardized methods for differentiating viruses from (non-virus) VLPs, inability to differentiate between infectious and non-infectious viruses, and insufficient sensitivity to detect low-abundance or smaller-sized viruses [25]. Some staining methods may also yield fluorescent colloids in virus-free wastewater matrices, resulting in background signals. FVM also requires startup costs akin to PCR methods, alongside specialized expertise for operation, maintenance, and analysis. While FVM exhibits potential as a surrogate parameter for virus monitoring, further studies are needed to define application boundaries in wastewater treatment systems.

This study aims to evaluate the potential of FVM analysis of VLPs to serve as an indicator for virus removal in wastewater treatment. We test the application of a previously optimized FVM protocol at three points in an active wastewater treatment and recycling facility. Considering the high diversity of viruses including numerous undescribed phages in wastewater, we implemented a non-targeted FVM approach to rapidly detect a broad suite of viruses [26,27,28]. We compare FVM results to culture-based detection of somatic and (F+) specific coliphage, two well-studied bacteriophages that infect *Escherichia coli* and are frequently used as indicators of fecal contamination [18]. We also compare VLP event counts enumerated by FVM with viral genome copies (gc) of three high-titer viruses: PMMoV, CrAss, and Tomato Brown Rugose Fruit Virus (ToBRFV) enumerated by digital droplet (dd)PCR. PMMoV and ToBRFV (both plant-infecting viruses) as well as CrAss (bacteria-infecting viruses) are passed through human stool and have been frequently detected at high concentrations in wastewater samples [21,29]. By comparing results from sampling points taken at different stages of treatment, we assess FVM as an indicator of total viral load and treatment efficiency. We discuss the strengths, limitations, and the next steps required to improve the use of FVM for treatment process monitoring.

## 2. Materials and Methods

### 2.1. Sample Collection and Processing

24 h composite wastewater samples were collected at three sampling points from the University of California, Davis Wastewater Treatment Plant (UCDWWTP) once per week for 11 weeks between May and July of 2023 (a total of 33 samples, 11 from each of the three sampling points). UCDWWTP treats wastewater from the University of California, Davis (UC Davis) campus which had an average flow rate of 1.3 MGD during the sampling period. This includes instructional buildings, laboratories, student housing, agriculture operations, and animal processing facilities, reflecting the university’s large agricultural and veterinarian programs [30]. Composite samples of wastewater-influent (INF), secondary-treated effluent (SEC), and tertiary-treated effluent (EFF) were collected using autosamplers that were maintained at 3 °C. The autosamplers collecting INF and EFF were flow-proportional, while the autosampler collecting SEC was time-based. The average daily sample volumes were 9.1 L for INF, 8.8 L for SEC, and 12 L for EFF.

After collection, UCDWWTP staff aliquoted the bulk samples into sterile polypropylene bottles (1 L for INF, 2 L for SEC and EFF) and stored the samples at 4 °C. The research team then transported the samples (on wet ice) 1.5 miles to the campus laboratory, where they were refrigerated at 4 °C until processing. All sample handling in the laboratory was conducted within a biosafety cabinet, and all processing was completed within 24 h of the UCDWWTP staff’s collection. Samples were vigorously shaken for 5–15 s before aliquoting for coliphage plaque assays, FVM, and molecular analysis by ddPCR.

### 2.2. (F+) Specific and Somatic Coliphage Plaque Assays

Coliphage enumeration followed EPA method #1602, (F+) specific and somatic coliphage in water by single agar layer (SAL) procedure [31]. Briefly, INF samples were diluted 100-fold and enumerated using the double agar layer (DAL) method as described in section 11 of EPA method 1602. This method involved spiking 500 μL of the diluted sample and 100 μL of the corresponding host bacteria (*E. coli* Famp for F+ coliphage and *E. coli* CN-13 for somatic coliphage), into 10 mL tubes containing 5 mL of molten 0.7% tryptic soy agar (TSA) “top agar”. The mixture was poured onto 1.5% TSA “bottom agar” plates and allowed to solidify. SEC and EFF samples were enumerated using the SAL method described in section 12 of EPA method 1602. This method involved adding 100 mL of sample and 10 mL of the corresponding host bacteria to 100 mL of molten 2X TSA in a 250 mL flask. The mixture was incubated in a water bath set to 45 °C for 3–10 min, pipetted into 10 Petri dishes per sample, and left to solidify. Preliminary experiments indicated that somatic coliphage concentrations were higher than (F+) specific coliphages in SEC samples; thus, SEC samples were diluted 10-fold for somatic coliphage enumeration but left undiluted for (F+) specific coliphage. EFF samples were undiluted for both coliphage types.

Antibiotics included in agar mixtures were: nalidixic acid for somatic coliphage and ampicillin/streptomycin for (F+) specific coliphage, as per EPA method 1602. Bacteriophages ϕX174 and MS2 were used as positive controls for somatic and (F+) specific coliphage, respectively, with Milli-Q water serving as the negative control. Following solidification, the plates were incubated for 16–24 h at 36 °C ± 1 °C. Plaques were manually enumerated using a lightbox, and coliphage concentrations were reported as PFU per 100 mL of wastewater. Somatic and (F+) specific coliphage results from different sampling points were compared to each other using Student’s t-test to determine if significant differences were observed between INF and SEC (as EFF did not have any detectable PFUs). Coliphage PFUs were compared to VLP counts enumerated by FVM at the same sampling point using Pearson’s product–moment correlation.

### 2.3. FCM Sample Preparation and Enumeration

On each sampling date, we analyzed four replicates of each wastewater sample, four replicate negative controls, and three aliquots (from each sampling point and PBS) spiked with the positive control containing T4 bacteriophage at different concentrations (as determined by plaque assay). All samples, standards, and controls were analyzed on the same 96-well plate, following the layout as described in Table S2 in the Supplemental Materials. Wastewater samples were prepared for FVM analysis by filtering 15 mL of each wastewater sample through 100 µm vinyl membrane filters (Thermo Fisher Scientific, Waltham, MA, USA) into sterile 125 mL glass Buchner flasks. Once filtered, samples were diluted 100-fold in sterile phosphate-buffered saline (PBS, 7.4 pH, Thermo Fisher Scientific, Waltham, MA, USA), stored in 2 mL polypropylene microcentrifuge tubes, and refrigerated at 4 °C until analysis was completed later that day. Following preparation of a sterile 96-well plate with the cleaning and rinsing solutions run between each sample (Figure S3), 90 μL of sample or blank was added to the appropriate well and spiked with 10 μL of 0.5 μm calibration beads (Bang laboratories, Inc., Fishers, IN, USA) as a size reference standard (or a mixture or T4 bacteriophage and various size calibration beads for positive controls). All samples and controls were then fixed with glutaraldehyde (Sigma-Aldrich, St. Louis, MO, USA) at a final concentration of 0.5% and stained with SYBR Gold at 2 × 10^–5^ times the sample volume at room temperature. In the present analytical protocol, this staining equated to adding 2 μL of SYBR Gold to the 100 μL sample after the SYBR Gold had been diluted 10^4^-fold in dimethyl sulfoxide according to the manufacturer’s specifications. For the first seven weeks of sampling (until 29/06/2023), we prepared the 96-well plates by hand, pipetting under a lit flame. After that point, plate preparation was completed using an automated pipetting system (epMotion 5073, Eppendorf, Hamburg, Germany).

FCM analysis was completed using a Novocyte 2070 V flow cytometer (Agilent Technologies, Inc., Santa Clara, CA, USA). Prior to each day of analysis, the instrument was thoroughly cleaned using automated cleaning cycles and running hypochlorite solutions and Milli-Q water through the instrument’s fluidics until the background event count was below 5 events per microliter when analyzing nuclease-free water at the instrument’s slowest run rate (10 μL/min). The flow cytometer was set to record events with a minimum threshold of 400 on green florescence intensity (530 ± 30 nm emission wavelength) height, filtering out events with green florescence values below the threshold), and samples were run at a 10 μL/min flow rate (120 µL/min was used to clean and rinse wells). Samples were analyzed using a 488 nm wavelength laser and measurements were recorded for forward-scatter, side-scatter, and green fluorescence intensity. Results are expressed as events per 100 milliliters of undiluted wastewater (events/100 mL wastewater).

### 2.4. FVM Method Validation and Quality Control

We applied several controls and size reference standards to support the validation of instrument parameters. For the infectious virus positive control, we spiked T4 bacteriophage (ATCC 11303-B4), a Myoviridae virus frequently used as a non-human infectious surrogate for enteric viruses. We also spiked fluorescent polystyrene calibration beads measuring 0.2, 0.5, and 0.8 μm in diameter (Bangs Laboratories, Inc., Fishers, IN, USA) as controls to indicate approximate particle size. T4 bacteriophage and its *E. coli* host (Migula) Castellani and Chalmers (ATCC 11303) were obtained from the American Type Culture Collection (ATCC) and propagated from freeze-dried specimens. High-titer T4 bacteriophage stocks were prepared as described by Safford et al. (2023), enumerated to be 1.7 × 10^12^ (±1.03 × 10^12^) PFU/100 mL by DAL, diluted 100-fold in sterile PBS, and frozen at −80 °C for use during the sampling period [11]. Prior to each week’s analysis, a new T4 aliquot was thawed, vortexed, and serially diluted to 10^9^, 10^8^, and 10^7^ equivalent PFUs/100 mL, vortexed with the calibration beads, and spiked into an aliquot of each sample as a positive control. We included PBS (pH 7.4) as sample blanks (negative controls). To facilitate future comparisons of this data with wastewater data collected from other wastewater treatment plants, likely using different flow cytometers, we also selected the 0.5 µm polystyrene bead as an internal control to be spiked in each sample. We ran cleaning wells comprised of 200 μL of Novoclean solution (Agilent Technologies, Inc., Santa Clara, CA, USA) followed by 200 μL of Milli-Q water between each sample. These solutions served the dual purpose of cleaning the instrument between each sample and checking for sample carryover.

Additional validation steps included the following: We enumerated decimal dilutions of T4 bacteriophage diluted with PBS (pH 7.4, Thermo Fisher Scientific, Waltham, MA, USA) by both FVM and DAL plaque assay and assessed correlations between the two detection methods. We seeded known concentrations of T4 bacteriophage into wastewater samples and compared the VLP event counts of the seeded samples to non-seeded samples and used the Student’s t-tests to determine if significant differences were observed. Additionally, we assessed the potential for the formation of non-viral artifacts (colloids of fluorescent stain) by performing the FVM protocol on grab samples of wastewater INF, SEC, and EFF that were both filtered (100 µm, Thermo Fisher Scientific, Waltham, MA, USA) and ultrafiltered (100 kDa MWCO, Amicon Ultra Centrifugal Filter, Millipore Sigma, St. Louis, MO, USA) prior to analysis.

### 2.5. FCM Data Management and VLP Gate

FCM data was recorded using the instrument’s proprietary software (NovoExpress 1.4.1). Analysis was completed using FlowJo V10.9, Microsoft Excel, and Python 3 via Jupyter Notebook Version 6.0.3. We established two-dimensional gates to differentiate VLP events from total events based on green fluorescence and side-scatter. To establish a VLP gate, we analyzed six isolated and purified bacteriophages ranging in genomic size from 13 to 170 kilobase pairs (Table S1) by FVM. The outer bounds of the FCM profile associated with each of the virus clusters were recorded and used to establish the gate associated with VLP events (Gate A). ϕ6 bacteriophage was generously provided by Dr. Sam L. Díaz-Muñoz at the University of California, Davis. Four additional phages were isolated from wastewater, characterized, and generously provided by Dr. Katrine Whiteson at the University of California, Irvine [32,33]. Gate A’s upper limits were set at 700 for side-scatter (height) and 10,000 for green fluorescence (height) with lower limits set by the instrument’s thresholds (0 for side-scatter and 400 for green fluorescence). A full list of viral targets including source, physical and genomic information, and outer bounds of their FCM profile are listed in Table S1 of the Supplemental Materials.

To assist in FCM profile characterization, we introduced a separate gate representing “high nucleic acid” (HNA) particles (Gate B) for particles with side-scatter values between 750 and 20,000 and green fluorescence values between 30,000 and 400,000. These particles are in a similar range to the HNA particles referenced by Huang et al. (2016) and Santos et al. (2019), with some adjustments to instrument sensitivity [10,34]. HNA particles are expected to include some bacteria, free-living amoebae, giant viruses, and other organisms with genome lengths larger than most bacteriophage and human enteric viruses [7,28]. HNA particles are relevant to wastewater treatment as they can be used to estimate bacterial-removal efficiency. Gates C2, C5, and C8 were included to encircle, respectively, the 0.2, 0.5, and 0.8 μm calibration beads spiked into the positive controls. Unless otherwise noted, averaged results from replicates are reported with associated standard deviation (±1 SD).

### 2.6. Tertiary Treatment Exploration with UV-Influent Samples

To further explore changes in event count between the SEC and EFF sampling points, we added a sampling location after the filtration stage of tertiary treatment but prior to UV treatment (PUV). For this investigation, 24 h composite samples were collected at three sampling points (SEC, PUV, and EFF) once per week for three weeks in July 2024 (nine total samples, with three from each sampling point). We used the same experimental conditions as the previous sampling campaign and included three positive controls, three negative controls, and one replicate of each sample from all sampling dates.

### 2.7. Molecular Analysis by ddPCR

RNA and DNA were concentrated, isolated, and extracted using the MagMAX™ microbiome ultra nucleic acid isolation kit (catalog number #A42357, Thermo Fisher Scientific, Waltham, MA, USA) following the procedure outlined by Muralidharan et al. (2024) [35]. Additional details related to the extraction protocol are provided in the Supplemental Materials. The ddPCR assay targeted PMMoV, CrAss, and ToBRFV in a multiplex format designed by combining previously validated qPCR protocols [36,37,38]. A full list of PCR primer and probe sequences can be found in Table S3 of the Supplemental Materials. Multiplex optimization involved assessing primer compatibility, running annealing temperature gradients (55–65 °C), evaluating assay interference, and determining the most effective level of dilution for each of the different sample types. From this protocol optimization, we found no issues with primer and probe compatibility and no significant assay interference. The ideal annealing temperature was determined to be 58 °C, and the dilution factors used were 100-fold for INF, 10-fold for SEC, and undiluted for EFF. Additional details regarding assay cycling times are provided in Table S4 of the Supplemental Material.

gBlock gene fragments (Integrated DNA Technologies, Coralville, IA, USA) containing the target viral sequences were used as positive controls for the multiplex assays, and their oligonucleotide sequences are displayed in Table S5 of the Supplemental Materials. All samples and reagents were stored at −80 °C and thawed on wet ice. Preparation took place within a sterile PCR hood, and nuclease-free water served as a no-template control (NTC). Reactions were comprised of 5.5 μL sample (diluted nucleic acid extract) and 16.5 μL of mastermix (one-step RT-ddPCR advanced kit for probes, Bio-Rad Laboratories, Hercules, CA, USA). The plates were then heat-sealed at 180 °C for 3 s, vortexed on each side for 5 s, and centrifuged at 115 RPM for 15 s [35]. Plates were then set in an auto-droplet generator (Bio-Rad Laboratories, Hercules, CA, USA), thermocycled at an annealing temperature of 58 °C, and loaded into a ddPCR machine (QX600, Bio-Rad Laboratories, Hercules, CA, USA) for overnight processing. ddPCR-enumerated target gc from different sampling points were compared to each other using a Kruskal–Wallis Test and the Dunn–Bonferroni post-test to determine if there was a significant difference between sampling points. Target virus genomes were also compared with VLP from the same sampling point (enumerated by FVM) using Pearson’s product–moment correlation.

### 2.8. Determination of Limits of Detection (LoD)

Limits of detection (LoD) for coliphage plaque assays were determined by the theoretical limit of detection for the analytical method (1 PFU per equivalent volume of wastewater analyzed). The LoD for ddPCR and FVM was determined following the procedure outlined by Armbruster and Pry (2008), which included analyzing 20 replicates of a blank sample to calculate the limit of blank (LoB) and 20 replicates of a low-concentration positive sample to establish the LoD [39]. Nuclease-free water was used as the blank, and the positive controls (gBlocks for ddPCR and T4 bacteriophage for FVM) were used as low-concentration positive samples to calculate the LoB and LoD. Equations (1) and (2) are used to calculate the LoB and LoD, respectively.

LoB = mean(blank) + 1.64 × SD (blank)(1)

LoD = LoB + 1.64 × SD (low concentration sample)(2)

Several dilutions of low-concentration positive samples were analyzed until the lowest concentration where no more than 5% of the values were less than the LoB was confirmed (establishing a 95% confidence interval for both LoDs). Because LoDs were established using the instrument’s raw data, ddPCR positive droplet counts were used to determine if samples were above the LoD, and then these values were converted to equivalent gc for reporting using the instrument’s software (QX Manager 2.1 Standard Addition, Bio-Rad Laboratories, Hercules, CA, USA). The LoD for each assay, as well as the equivalent volume of wastewater analyzed per replicate are listed in Table 1. The limits of detection listed are instrument and assay-specific and could potentially be reduced by implementing more highly purified blank samples, more efficient instrument maintenance and calibration, or adjusting sample volumes.

## 3. Results and Discussion

### 3.1. FVM Method Validation

The FVM protocol applied in this study was based on an optimized protocol for the non-targeted analysis of VLPs based on the detection of T4 suspended in aqueous solutions [11]. Adaptations for wastewater analysis included using SYBR Gold instead of SYBR Green (to enumerate both RNA and DNA viruses) and omitting heating to 37 °C (to maintain the high throughput and rapid analysis characterized by online monitoring). The adapted parameters were successfully applied in the original study but were not optimal for T4 enumeration. To validate the adapted method for wastewater, we spiked T4 into both PBS and wastewater at three dilution levels. We observed a strong positive correlation (r = 0.99, df = 1, *p* < 0.05) between VLP event counts enumerated by FVM of the T4 positive control spiked into PBS and PFU enumerated by DAL assay (Table 2). When wastewater was seeded with T4 at its highest concentration (~10^8^ PFU/100 mL), VLP counts increased significantly in each sample (Table 3). T4 seeded at lower titers into wastewater samples were masked by the high titer of VLPs present in wastewater (Figure S4).

We also evaluated the potential for fluorescence artifacts (colloids of the stain) to form in “virus-free” wastewater matrices. We removed viruses (>100 kDa) from the background wastewater matrix via ultrafiltration. When spiked with submicron beads or T4 bacteriophage as a positive control, the FVM signals generated by the positive control were clearly distinguishable in the virus-free wastewater matrices (Figure S5). Counts of VLPs in un-spiked, stained virus-free wastewater were low and a similar order of magnitude results as for PBS, indicating negligible formation of colloids from stain interactions with the matrices. Stain type, concentration, additives, and incubation temperature as well as sample matrix are likely to influence colloid formation. Dlusskaya et al. (2021) observed colloid formation of fluorescent stains in ultrafiltered wastewater (yielding non-virus signals that appeared as VLPs) [25]. The present study applied SYBR Gold stain with incubation at room temperature while Dlusskaya et al. (2021) utilized SYBR Green and 80 °C incubation [25]. We reiterate others in emphasizing the importance of using appropriate controls when applying FVM protocols. Our results offered sufficient evidence of the detection of intact viruses in the complex wastewater matrix using FVM to proceed with its application.

### 3.2. Application of FVM Protocol for Wastewater Monitoring

**Figure 1:**
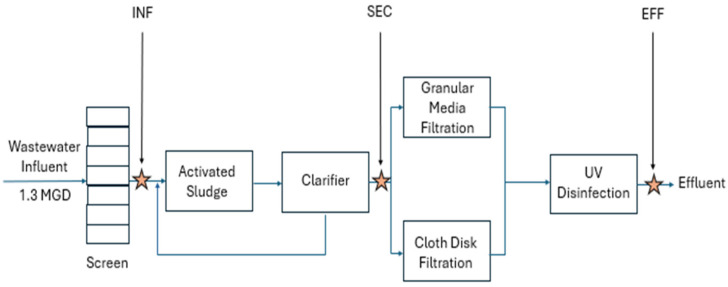
Simplified schematic of the UCDWWTP treatment train process and sampling points of this study (labeled and denoted with a star).

**Figure 2:**
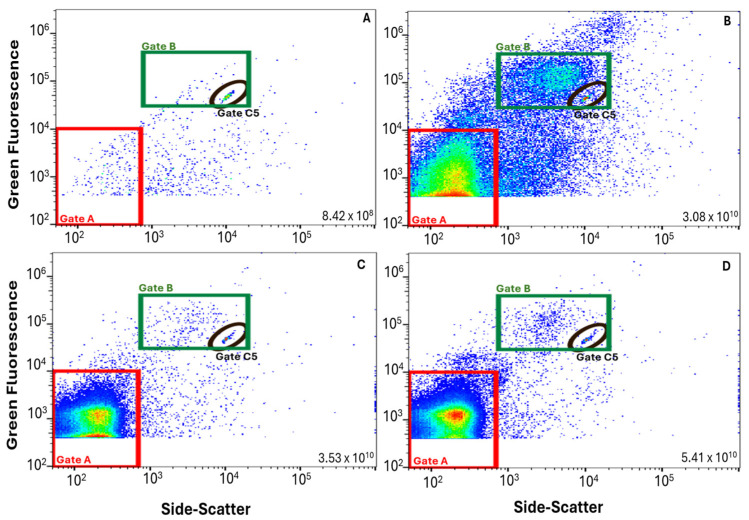
Sample data (10/05/2023) example demonstrating features of FCM profiles. All samples were spiked with 0.5 μm calibration beads (Gate C5, black oval). Gate A (red) represents events labeled as VLPs and Gate B (green) represents HNA particles. The total event count (per 100 mL) is listed in the lower right-hand corner of each subplot. Subplot labels: (**A**) negative control (sterile PBS), (**B**) Wastewater-influent (INF), (**C**) secondary-treated effluent (SEC), and (**D**) tertiary-treated effluent (EFF).

**Figure 3:**
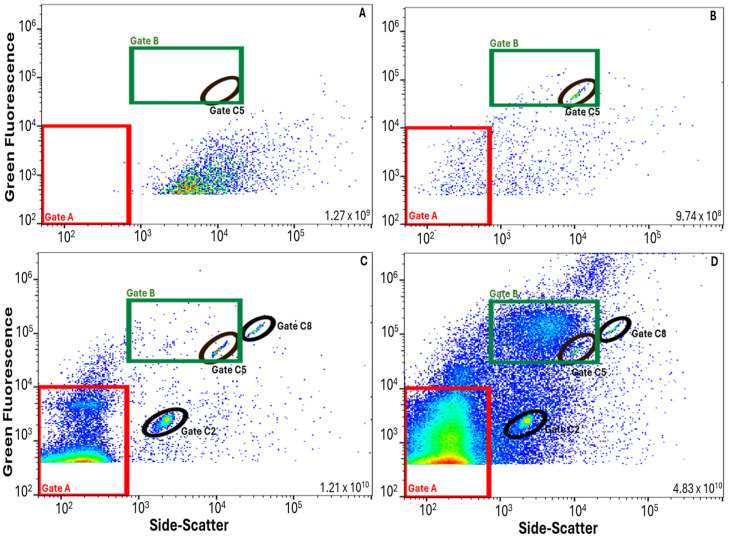
Raw FVM data (10/05/2023 samples) displayed as two-dimensional scatterplots where the density of events (relative to the total number of events in the sample) is displayed using colors from dark to bright (dark blue representing events in relatively low-density and bright red representing events in relatively high-density). Gate A (red) represents virus-like particles (VLPs), Gate B (green) represents high nucleic acid (HNA) particles, and Gates C2, C5, and C8 (black ovals) indicate spiked fluorescence polystyrene calibration bead controls (for 0.2, 0.5, and 0.8 μm bead sizes, respectively). The total event count (per 100 mL) is listed in the lower right-hand corner of each subplot. Subplot labels: (**A**) unstained wastewater-influent with no spike, (**B**) negative sample (PBS) stained and spiked with 0.5 μm beads, (**C**) negative sample (PBS) stained and spiked with T4 bacteriophage and spiked with all three calibration beads (0.2, 0.5, and 0.8 μm), and (**D**) wastewater-influent stained and spiked with T4 bacteriophage and all three calibration beads (0.2, 0.5, and 0.8 μm).

The wastewater treatment plant evaluated in this study includes an oxidation ditch (activated sludge) with disc aerators, followed by clarification, filtration (parallel cloth disc and mixed media filters), and UV disinfection prior to recycling or discharge (Figure 1). Application of the FVM protocol to wastewater samples revealed unique microbial characteristics from each of the three sampling points. By visual inspection, the FCM profiles taken at each sampling point were generally consistent throughout the sampling period when displayed as two-dimensional dot plots with green fluorescence and side-scatter (Figure 2). The density of events (relative to the total number of events in the sample) is displayed using colors from dark to bright (dark blue representing events in relatively low-density and bright red representing events in relatively high-density). INF samples exhibited two primary clusters, quantifiable within Gates A and B. The particles in Gate A (79% of total INF events) are considered VLPs, while particles in the region of Gate B are considered HNA particles, this is reflected in the two-dimensional plots comparing un-spiked samples to samples spiked with T4 bacteriophage and submicron beads (Figure 3). The SEC and EFF samples generally did not exhibit distinct HNA clusters. SEC samples had a similar number of total events as INF samples, but a larger percentage of VLP events (98%). EFF had the largest number of total events of which 99% were categorized as VLP events. Total and VLP event counts demonstrated a significant positive relationship (r = 0.99, degrees of freedom (df) = 9, *p* < 0.001) at all three sampling points. Table S6 of the Supplemental Materials displays a summary of the FCM data collected, including total and VLP event counts.

**Figure 4:**
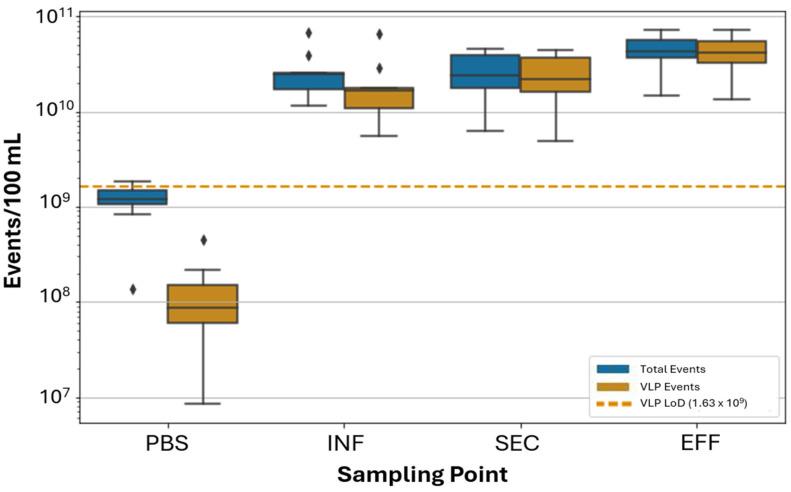
FVM total and VLP daily (median of four replicates) event counts taken weekly for the negative control (PBS), wastewater-influent (INF), secondary-treated effluent (SEC), and tertiary-treated effluent (EFF) taken across the sampling period (*n* = 11). Total event counts include spiked 0.5 μm calibration beads, which accounted for 5.87 × 10^7^ (±5.87 × 10^7^) event/100 mL in PBS. Data points denoted with “⧫” indicate outliers, defined as values more than 1.5× the interquartile range beyond the first or third quartile.

On average, EFF exhibited the highest total event counts, followed by INF, then SEC (Figure 4). The daily median (of four replicates) event counts per 100 mL of wastewater ranged between 1.14 × 10^10^ and 6.81 × 10^10^ for INF, 6.19 × 10^9^ and 4.53 × 10^10^ for SEC, and 1.46 × 10^10^ and 7.27 × 10^10^ for EFF (including the 0.5 μm calibration bead). For VLP events, EFF also had the highest average count, followed by SEC, then INF. The daily median (of four replicates) VLP event counts per 100 mL of wastewater ranged between 5.46 × 10^9^ and 6.63 × 10^10^ for INF, 4.95 × 10^9^ and 4.39 × 10^10^ for SEC, and 1.35 × 10^10^ and 7.10 × 10^10^ for EFF. PBS (negative control) had an average total event count of 5.45 × 10^8^ and an average VLP count of 1.13 × 10^8^ events/100 mL.

**Figure 5:**
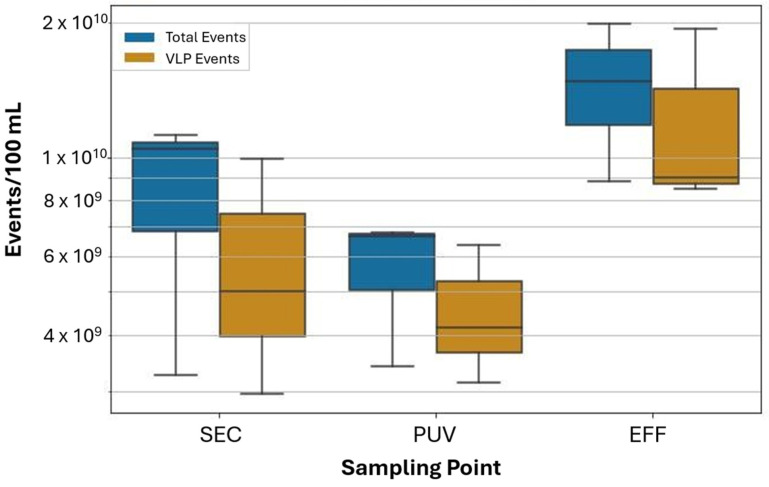
FCM total and VLP event counts from secondary-treated effluent (SEC), UV-influent (PUV), and tertiary-treated effluent (EFF) taken in July of 2024 (*n* = 3). Total event counts include spiked 0.5 μm calibration beads which accounted for 5.87 × 10^7^ (±5.87 × 10^7^) events/100 mL in PBS.

The distinct FCM profile characteristics observed at different sampling points highlight key changes in the microbial community through the wastewater treatment process. The presence of a clear HNA cluster in the INF, and its absence in the SEC, highlights efficient removal of bacteria and larger microorganisms during secondary treatment. To assess whether the increase in VLP counts from the SEC to the EFF sampling point occurred following the filtration or UV disinfection steps, we analyzed three samples taken between the filtration and UV disinfection steps and compared them with samples from SEC and EFF taken on the same day (Figure 5). VLP counts for SEC and EFF, taken concurrently were within the range of previous measurements, with 5.94 × 10^9^ (±5.01 × 10^9^) VLP events for SEC and 1.23 × 10^10^ (±8.98 × 10^9^) VLP events for EFF per 100 mL of wastewater. PUV samples were enumerated at 4.55 × 10^9^ (±4.16 × 10^9^) VLP events per 100 mL of wastewater.

FVM analysis of samples collected immediately before and immediately after filtration (SEC and PUV, respectively) show a decrease in VLP counts following filtration. The decrease in VLPs was smaller than that of total event counts, which is consistent with our expectation that cloth and media filtration are less effective at removing small particles, such as viruses and cellular detritus, compared to larger particles like bacteria and suspended solids. Analysis of samples collected before and after UV disinfection (PUV and EFF, respectively) revealed a large increase in VLPs following UV disinfection. While UV disinfection efficiently inactivates microorganisms, it primarily damages virus genetic material (free-floating or encapsulated nucleic acids) and inactivated virus particles are likely to remain detectable by FVM [40]. We postulated that photolytic degradation of nucleic acids, with virus particles remaining intact, may lead to a decline in fluorescence intensity but a similar number of particles after UV treatment. The increase in VLP counts could be an artifact of increased free nucleic acids or other cellular detritus released in the water (from viral or non-viral sources) by the decay of microbes after disinfection. Protein-RNA cross-links initiated by UV exposure may also lead to capsid damage or additional nucleic acid damage, further contributing to changes in fluorescent staining efficiencies [41]. Future studies designed specifically to assess the contribution of non-virus artifacts to the FVM signal and delineate non-virus biological particles from intact virus particles would be valuable, particularly in monitoring the performance of disinfection processes.

### 3.3. Comparison of VLPs by FVM with Somatic and F+ Specific Coliphage

**Figure 6:**
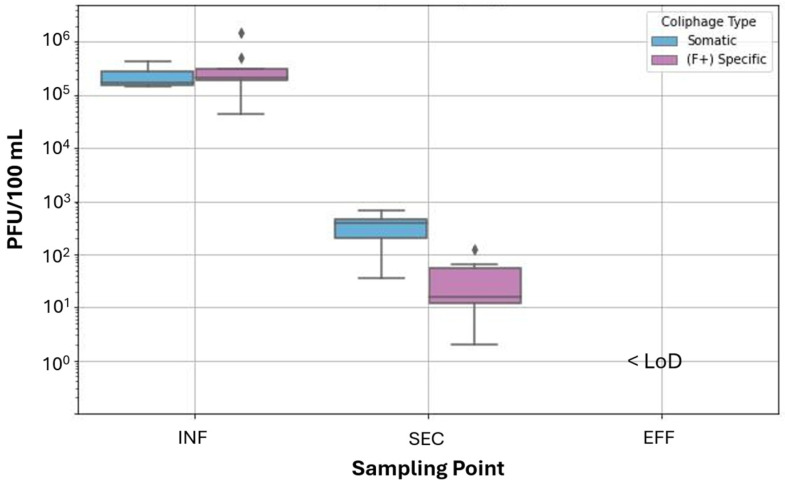
Coliphage PFU per 100 mL wastewater for wastewater-influent (INF), secondary-treated effluent (SEC), and tertiary-treated effluent (EFF) across the sampling period. EFF values were below the theoretical LoD (≤1 PFU per 100 mL). Data points denoted with “⧫” indicate outliers, defined as values more than 1.5× the interquartile range beyond the first or third quartile.

Somatic and (F+) specific coliphage enumerated by culture-based plaque assays demonstrated, as expected, efficient virus removal via physical treatment processes and disinfection. Somatic and (F+) specific coliphage data were recorded for nine and seven sampling days, respectively, due to technical issues with the assay (Figure 6). The mean concentration of somatic coliphage was 2.28 × 10^5^ (±9.96 × 10^4^) PFU/100 mL for INF and 3.59 × 10^2^ (±2.02 × 10^2^) PFU/100 mL for SEC. The mean concentration of (F+) specific coliphage was 3.70 × 10^5^ (±4.30 × 10^5^) PFU/100 mL for INF and 3.69 × 10^1^ (±4.14 × 10^1^) PFU/100 mL for SEC. Both assays resulted in no detectible PFUs in EFF and the assay had a theoretical LoD of 1 PFU per 100 mL of wastewater.

Coliphage plaque assays provide a targeted approach to assess viral treatment efficiency. The observed 2.8 log removal of somatic coliphage and 4 log removal of (F+) specific coliphage from INF to SEC, and greater than 5 log removal for both targets from INF to EFF, demonstrates effectiveness in the treatment process. While plaque assays are selective and do not reflect total virus abundance in wastewater, they remain valuable for assessing virus inactivation. We expected that results for the inactivation of infectious coliphage via UV disinfection would likely deviate from VLP event counts enumerated by FVM since the FVM assay implemented does not distinguish between infectious and non-infectious viruses. Rather, the FVM assay applied herein quantifies all particles stained with nucleic acid-binding dyes or exhibiting autofluorescence above the instrument’s threshold. Indeed, coliphage was significantly removed or inactivated from INF to SEC and EFF, while VLP counts increased significantly overall from INF to EFF. While VLP counts decreased from SEC to PUV samples (through filtration, a physical removal process), the increase in VLP counts following UV disinfection was more substantial than we anticipated. Results of the Student’s t-test analysis comparing coliphage PFU at different sampling points as well as Kruskal–Wallis test and the Dunn–Bonferroni post-test for VLP counts during the 11 week sampling period are included in Tables S7 and S8, respectively.

We also postulated that results for coliphage titers collected within a sampling location would correlate with VLPs measured in the same samples. Coliphage assay results for INF and SEC sampling locations are plotted against FVM VLPs in Figure S1 of the Supplemental Materials. Correlation coefficients between coliphage PFUs and FVM VLPs within a given wastewater treatment step were not statistically significant (*p* > 0.1). The direction of the relationships was also inconsistent. For instance, the correlation coefficient between VLPs and somatic coliphage was negative in INF (r = −0.37, df = 7) and positive in SEC (r = 0.46, df = 8). A negative correlation coefficient was found between VLPs and (F+) specific coliphage in both the INF (r = −0.55, df = 7) and SEC (r = −0.38, df = 6).

While somatic and (F+) specific coliphage are removed and inactivated through secondary treatment, other bacteriophage may propagate throughout the activated sludge process [42]. Cellular lysis and microbial decay also induce the formation of non-encapsulated nucleic acids, extracellular vesicles, and other cellular debris whose small size and variable surface properties lead to low removal rates during sedimentation [43,44]. The introduction of these particles throughout treatment may increase VLP counts by producing FVM signals that are similar to viruses [2,24]. Regardless, the poor correlations between VLP and coliphage removal, including the increase in VLP event counts following UV disinfection, reinforce that FVM using SYBR Gold staining was not a reliable indicator of infectious coliphage titers or inactivation in the present study. Exploration of improved particle-gating strategies for FVM by better utilizing the multi-dimensional data outputs and clustering methods may provide value in refining virus clusters from other particle types [11]. Co-labeling virus particles with nucleic acid stain and capsid or envelope protein tags have also shown promise in differentiating intact from non-viable viruses in FVM analyses [45,46].

### 3.4. Comparison Between FVM and ddPCR

**Figure 7:**
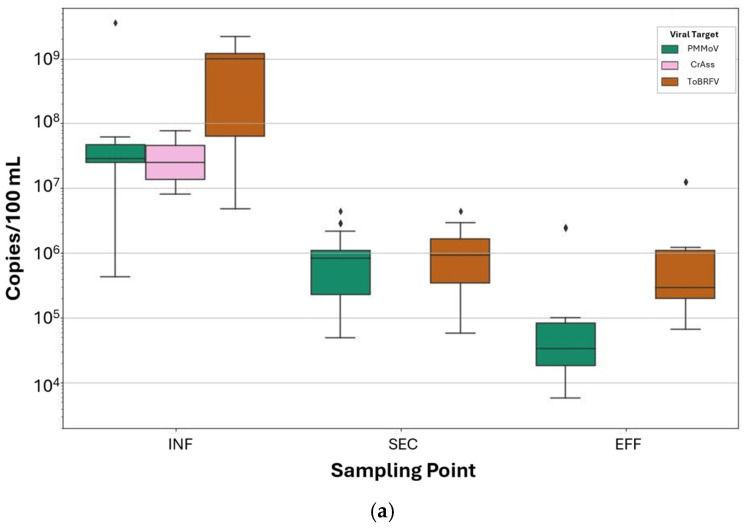

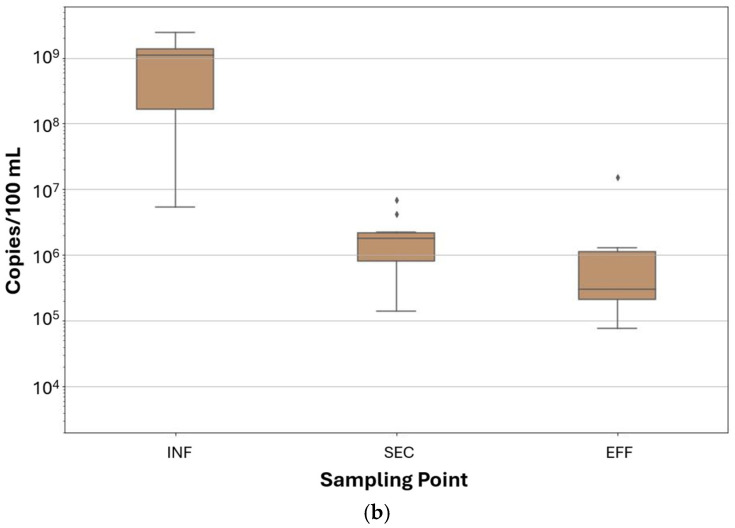
ddPCR viral target concentrations (gene copies/100 mL wastewater) at wastewater-influent (INF), secondary-treated effluent (SEC), and tertiary-treated effluent (EFF) sampling points for (**a**) Pepper Mild Mottle Virus (PMMoV), CrAssphage (CrAss), and Tomato Brown Rugose Fruit Virus (ToBRFV); and (**b**) combined ddPCR viral target (PMMoV, CrAss, and ToBRFV) concentration (gene copies per 100 mL wastewater). Data points denoted with “⧫” indicate outliers, defined as values more than 1.5× the interquartile range beyond the first or third quartile.

We analyzed 32 wastewater samples from the 11 week sampling in 2023 by ddPCR in triplicate with a multiplex assay targeting PMMoV, CrAss, and ToBRFV (Figure 7). Two EFF samples were unable to be analyzed due to issues during sample storage. ToBRFV had the highest concentration (gc/100 mL wastewater) at all three sampling points, followed by PMMoV, and CrAss. INF had the highest concentration for all three targets with daily mean (of three replicates) values ranging between 5.44 × 10^6^ and 2.13 × 10^9^ gc/100 mL wastewater. Daily mean viral target concentrations for SEC were between 6.65 × 10^4^ and 3.44 × 10^6^, and for EFF between 6.27 × 10^4^ and 1.26 × 10^7^ gc/100 mL wastewater. We also calculated the sum of viral target copies as a closer representation of virus load (for the selected suite of viruses). The mean concentration of the combined viral targets was 9.94 × 10^8^ (± 8.30 × 10^8^), 2.14 × 10^6^ (± 1.92 × 10^6^), and 2.31 × 10^6^ (± 4.84 × 10^6^) for INF, SEC, and EFF, respectively. ddPCR assay results are plotted against FVM VLPs in Figure S2. Correlation coefficients between ddPCR targets and VLPs within a treatment step, while frequently positive, were not significant.

We postulated that FVM results would correlate more strongly with ddPCR results than culture-based assays as both ddPCR and FVM methods rely on fluorescent-based labeling of nucleic acids. However, the overall trends between VLP events and ddPCR results once again diverged across sampling points (VLPs increased from INF to EFF while gc decreased overall). PMMoV and ToBRFV were detected at high concentrations and showed an overall decrease (as expected) from influent to effluent. PMMoV and ToBRFV remained consistently detectable in EFF but did not increase in concentration relative to SEC. Results of the Kruskal–Wallis test and the Dunn–Bonferroni post-test which compare ddPCR gc at different sampling points are included in Tables S9–S11 for PMMoV, ToBRFV, and total detected genome copies, respectively.

ddPCR offers precise estimates of target viral genome abundance through sequence-specific enumeration of nucleic acids. In contrast, the FVM assay broadly detects nucleic acids (including those in bacteria, viruses, vesicles, protozoa, and extracellular debris) due to the non-specific binding properties of SYBR Gold stain [47,48]. The discrepancies observed between ddPCR and FVM results suggest that FVM captures patterns in nucleic acid prevalence that are not represented by the high-titer viruses PMMoV, CrAss, and ToBRFV. Targeted FVM methods, for example, those applying fluorescence in situ hybridization (flow-FISH), may offer a more accurate representation of virus removal for targets of interest.

## 4. Conclusions

The results of this study highlight FVM’s ability to detect viruses in isolation and wastewater, but interpretation of FVM results when analyzing wastewater treatment processes remains challenging. We successfully defined a gating region for VLPs using a suite of bacteriophage isolated from wastewater, improving upon previous VLP-gating approaches and offering a transferable strategy for other laboratories. Additional improvements in multi-dimensional particle-gating strategies are likely to further enhance differentiation of particle types. We used T4 as an intact virus control and multiple sub-micron beads as size references to further enhance the transferability and reproducibility of the method applied.

We observed a strong positive correlation between isolated T4 bacteriophage PFU enumerated by culture-based assay and VLP enumerated by FVM, indicating a VLP event count 2 log higher than PFU, similar to the results reported from prior studies. We also reliably detected seeded intact bacteriophage when spiked at a concentration above the VLP signal generated by the wastewater. However, VLPs were found to increase through the wastewater treatment process, especially following UV disinfection, while somatic and (F+) specific coliphage exhibited >5 log-removal. These results indicate that non-specific FVM does not serve as a useful indicator for viral treatment efficiency in this context.

FVM can provide unique information and characterization as well as detect shifts in microbial populations, underscoring its potential as a complementary tool in process and environmental monitoring. Combining non-specific nucleic acid stains like SYBR Gold with specific protein or genetic stains could enhance FVM’s ability to distinguish viral particles from other nucleic acid-containing particles in wastewater but would likely increase time to results delivery and require manual sample manipulations. Establishing appropriate quality control and well-defined application boundaries will be critical as flow cytometry and virometry continue to play an increasing role in advancing water and wastewater treatment.

## Figures and Tables

**Table 1:** Sample assays employed with equivalent wastewater volumes tested for each assay, the number of replicates tested for each sample collected, and the limit of detection (LoD) determined.

Assay (Target)	Sampling Point	Equivalent Wastewater Volume	# Replicates	LOD
Somatic Coliphage (PFU)	INF	5 μL	4	2 × 10^4^ PFU/100 mL
	SEC	10 mL	1	1 × 10^1^ PFU/100 mL
	EFF	100 mL	1	1 × 10^0^ PFU/100 mL
(F+) Specific Coliphage (PFU)	INF	5 μL	4	2 × 10^4^ PFU/100 mL
	SEC	100 mL	1	1 × 10^0^ PFU/100 mL
	EFF	100 mL	1	1 × 10^0^ PFU/100 mL
Flow Virometry (VLPs)	INF	0.22 µL	4	1.63 × 10^9^ VLP/100 mL
	SEC	0.22 µL	4	1.63 × 10^9^ VLP/100 mL
	EFF	0.22 µL	4	1.63 × 10^9^ VLP/100 mL
ddPCR (PMMoV)	INF	2.78 µL	3	5.54 × 10^5^ gc/100 mL
	SEC	27.8 µL	3	5.54 × 10^4^ gc/100 mL
	EFF	278 µL	3	5.54 × 10^3^ gc/100 mL
ddPCR (CrAss)	INF	2.78 μL	3	6.81 × 10^5^ gc/100 mL
	SEC	27.8 μL	3	6.81 × 10^4^ gc/100 mL
	EFF	278 μL	3	6.81 × 10^3^ gc/100 mL
ddPCR (ToBRFV)	INF	2.78 μL	3	4.68 × 10^5^ gc/100 mL
	SEC	27.8 μL	3	4.68 × 10^4^ gc/100 mL
	EFF	278 μL	3	4.68 × 10^3^ gc/100 mL

**Table 2:** T4 (positive control) VLP event counts enumerated in buffer (PBS) by FVM compared to the equivalent PFU enumerated by double agar layer assay.

VLP Events/100 mL (*n* = 11, ±1 SD)	PFU/100 mL (*n* = 2)
3.2 × 10^10^ (±3.5 × 10^10^)	~1.3 × 10^8^
2.8 × 10^9^ (±3.1 × 10^9^)	~1.3 × 10^7^
5.0 × 10^8^ (±5.4 × 10^8^)	~1.3 × 10^6^

**Table 3:** Comparison of VLP event counts for samples with and without T4 spike. Mean and standard deviation (SD) values were reported as VLP events/100 mL.

Sample	Un-Spiked Mean (±1 SD)	Spiked Mean (±1 SD)	T-Statistic	*p-Value*
PBS (control)	1.27 × 10^8^ (±1.24 × 10^8^)	3.20 × 10^10^ (±3.53 × 10^10^)	−3.00	0.013
INF	1.94 × 10^10^ (±1.68 × 10^10^)	7.86 × 10^10^ (±6.95 × 10^10^)	−3.01	0.013
SEC	2.49 × 10^10^ (±1.41 × 10^10^)	7.64 × 10^10^ (±8.13 × 10^10^)	−2.13	0.059
EFF	4.3 × 10^10^ (±1.74 × 10^10^)	9.60 × 10^10^ (±7.39 × 10^10^)	−2.56	0.029

## Data Availability

Summative data are available in the supplementary materials. Additional data collected has been made available at: https://github.com/meljohnson9/Flow_virometry_in_wastewater_monitoring-MDPI_Viruses_2025-datasets (accessed on 6 January 2025).

## Supplementary Materials

The following supporting information can be downloaded at: https://www.mdpi.com/article/10.3390/v17040575/s1, Figure S1: FVM and coliphage correlation plot; Figure S2: FVM and ddPCR correlation plot; Figure S3: correlation coefficient matrix of results across sampling period; Figure S4: Mean VLP event counts per 100 mL of wastewater samples and PBS, with and without T4 bacteriophage; Figure S5: Flow cytometry plots of PBS and wastewater samples used for FVM method validation; Table S1: Isolated bacteriophage targets and their respective FCM profile ranges; Table S2: FVM 96-well plate setup used for FVM analysis; Table S3: ddPCR viral target oligonucleotide sequences; Table S4: ddPCR configuration and cycling times; Table S5: ddPCR viral target gBlock sequences; Table S6: Summary of results across duration of the sampling period; Table S7: Coliphage assay results comparison between INF and SEC using Student’s t-test; Table S8: FVM results of Kruskal–Wallis test and Dunn–Bonferroni post-test for VLP event counts; Table S9: ddPCR results of Kruskal–Wallis test and Dunn–Bonferroni post-test for PMMoV; Table S10: ddPCR results of Kruskal–Wallis and Dunn–Bonferroni post-test for ToBRFV; Table S11: ddPCR results of Kruskal–Wallis and Dunn–Bonferroni post-test for total detected gene copies; S1: Nucleic acid extraction protocol.
