# Supplementary material for: Flow Virometry in Wastewater Monitoring: Comparison of Virus-like Particles to Coliphage, Pepper Mild Mottle Virus, CrAssphage, and Tomato Brown Rugose Fruit Virus
Source: Viruses. 2025 Apr 16;17(4):575. doi: 10.3390/v17040575 (PMC12031537; doi:10.3390/v17040575)
Supplement: Supplementary file 1 [file viruses-17-00575-s001.zip › viruses-3439971-supplementary.pdf]

Supplemental Materials

for

Flow Virometry in Wastewater Monitoring: Comparison of Virus-like Particles to Coliphage, Pepper Mild Mottle Virus, CrAssphage, and Tomato Brown Rugose Fruit Virus

Melis M. Johnson, C. Winston Bess, Rachel Olson and Heather N. Bischel *

Department of Civil and Environmental Engineering, University of California Davis, Davis 95616, CA, USA; meljohnson@ucdavis.edu (M.M.J.)
* Correspondence: hbischel@ucdavis.edu

Contents: Five figures, eleven tables, and nucleic acid extraction protocol.

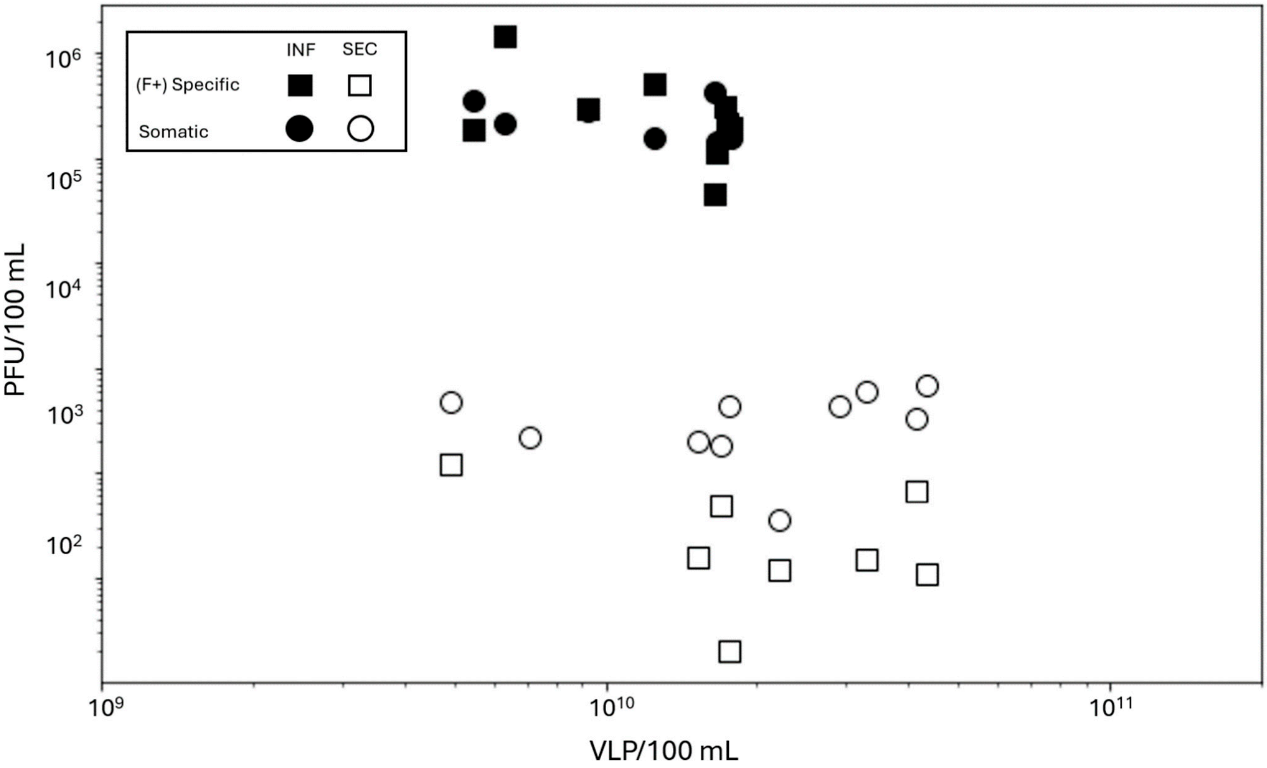

Figure S1. Virus like particles (VLP) enumerated by flow virometry (FVM) in wastewater influent (INF) and secondary treated wastewater (SEC) compared to plaque forming units (PFU) enumerated by culture assays for somatic and (F+) specific coliphage.

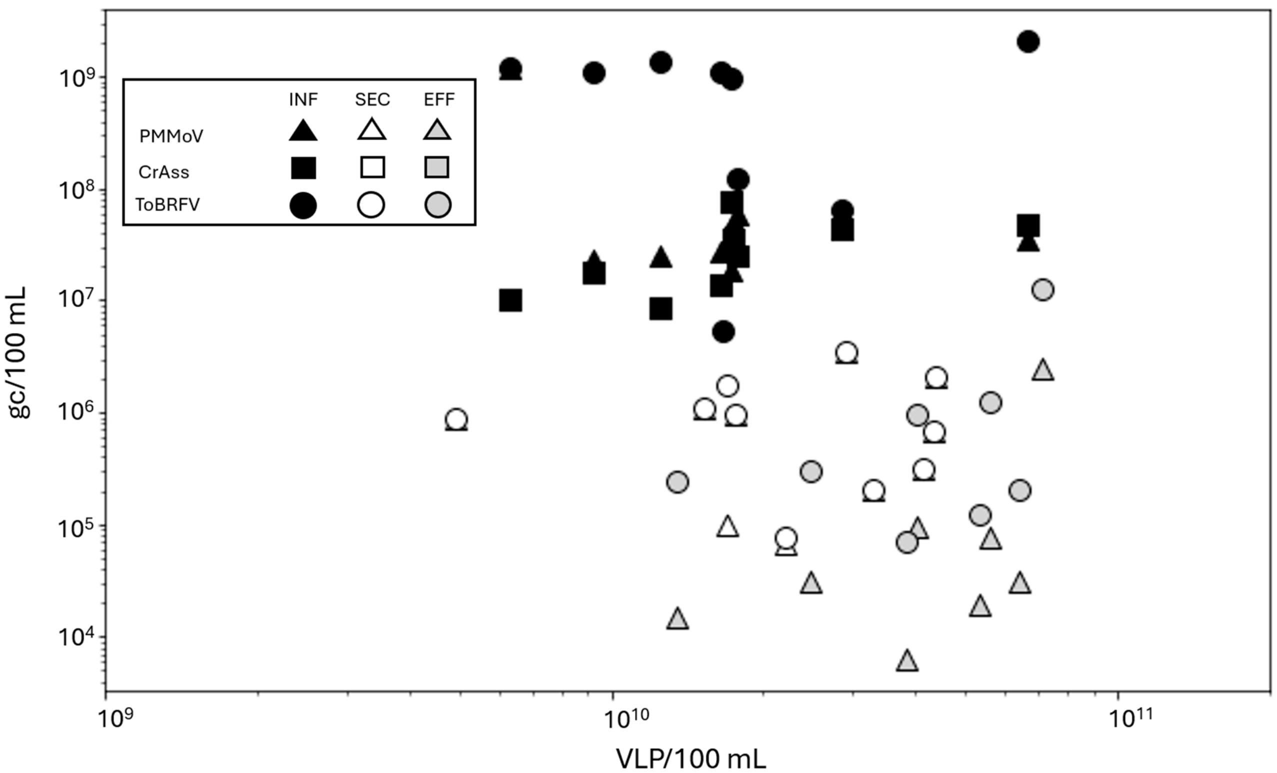

Figure S2. Virus like particles (VLP) enumerated by flow virometry (FVM) in wastewater influent (INF) and secondary treated wastewater (SEC) compared to genome copies (gc) enumerated by droplet digital (dd)PCR for three viruses (PMMoV, CrAss, and ToBRFV).

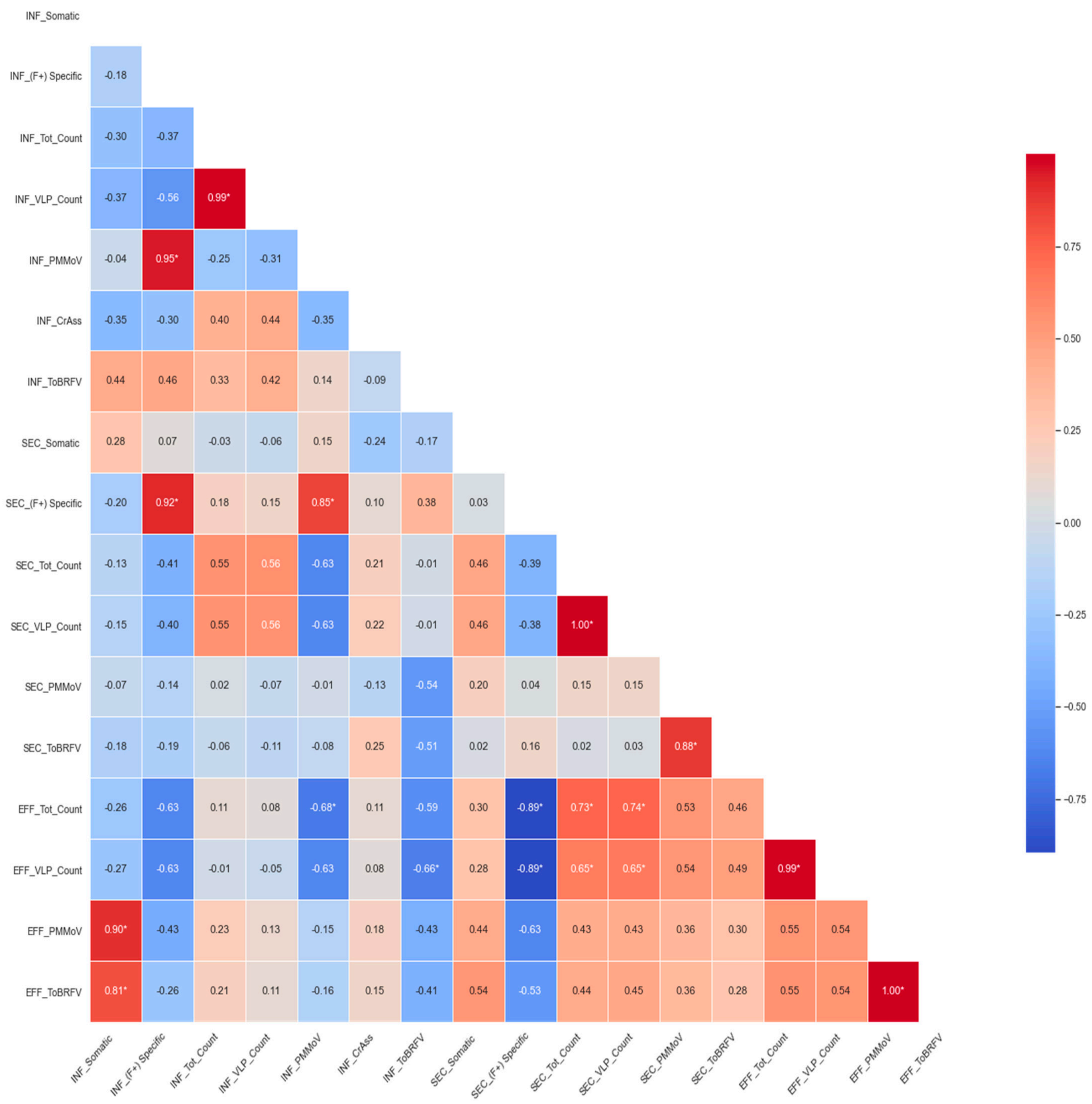

Figure S3. Correlation coefficient matrix for results across the 11-week sampling period where the Pearson correlation coefficient (r) is indicated in the square for each combination of results (on the x and y-axis), and "*" denotes that the correlation was considered to be statistically significant (p-value < 0.05).

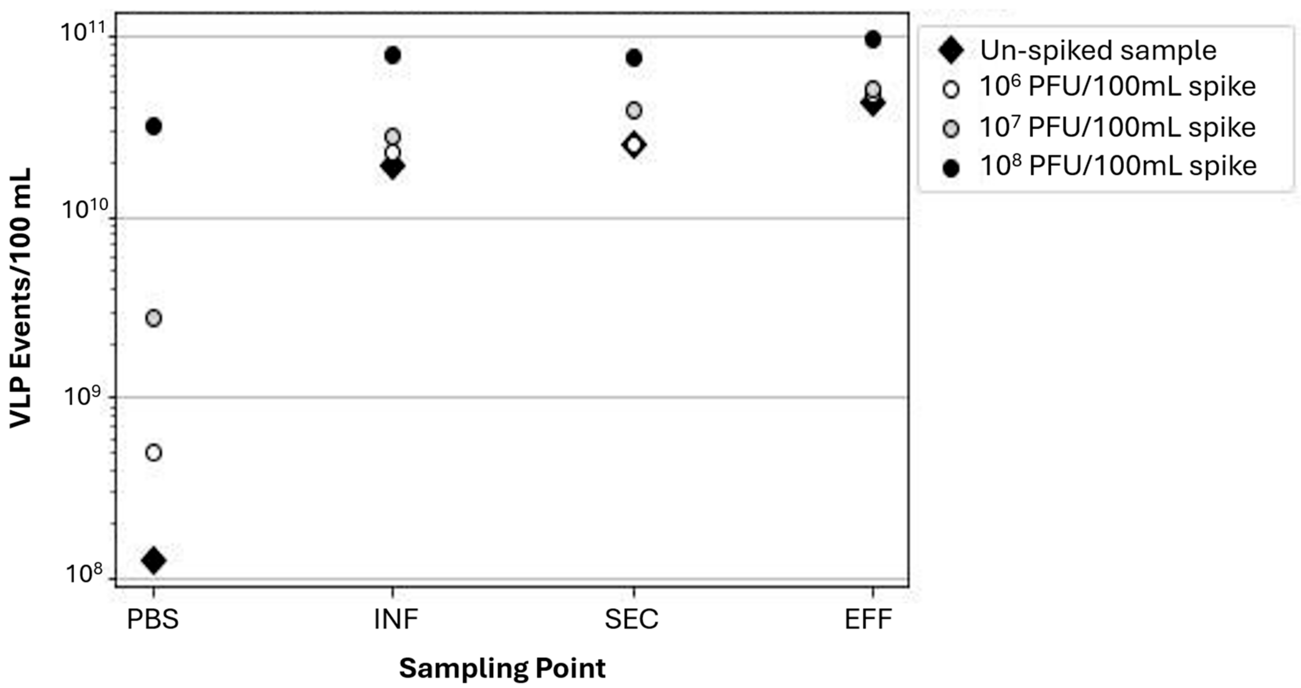

Figure S4. Mean VLP event counts per 100 mL of wastewater samples and PBS (n=11 per sample) with and without T4 bacteriophage spiked at three concentrations (measured by double layer plaque assay).

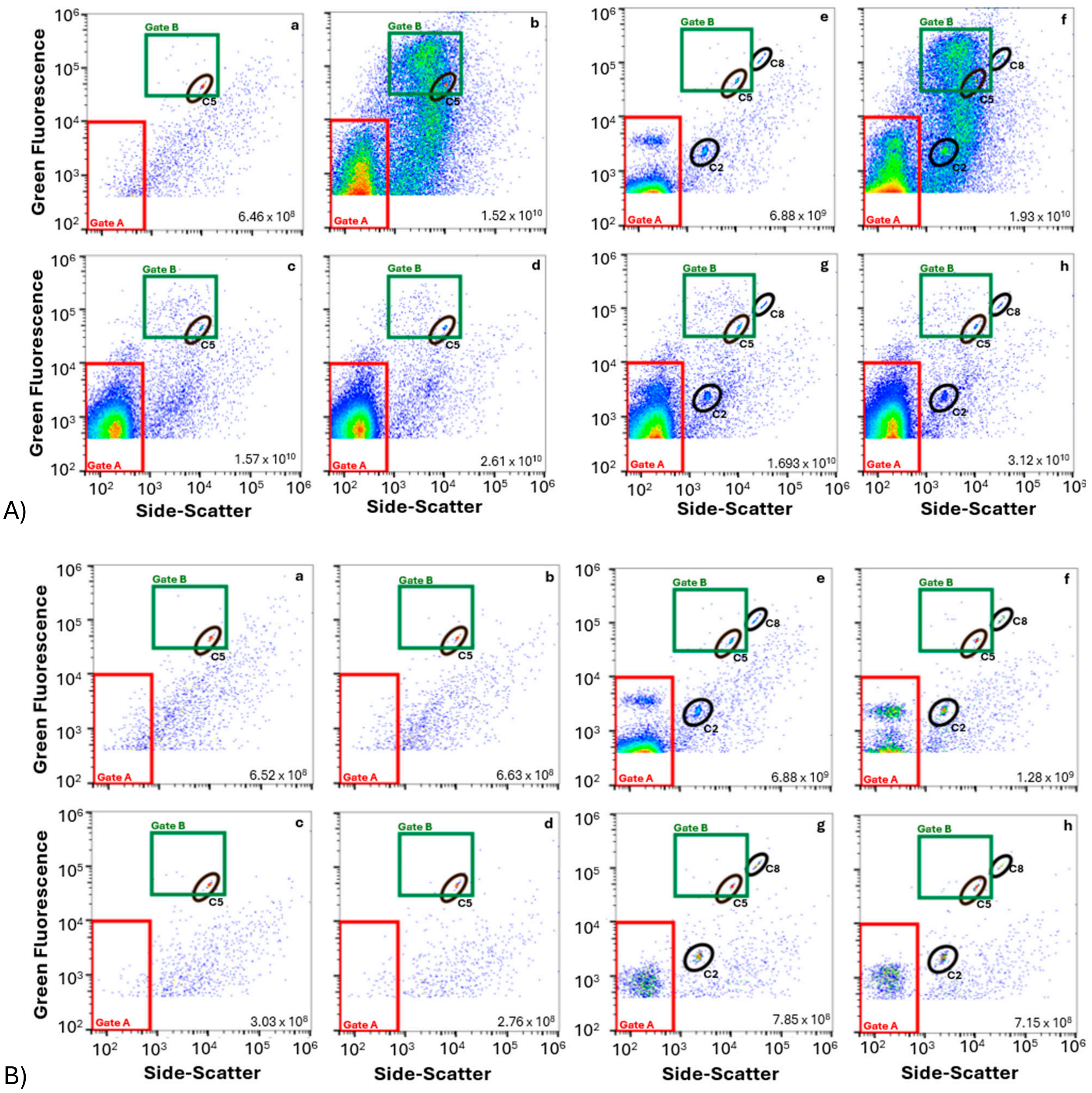

Figure S5. Flow Cytometry plots of PBS and wastewater samples used for FVM method validation. A) Wastewater samples filtered (100 µm pore size). B) Wastewater sample filtered (100 µm pore size) and ultrafiltered (100 kDa MWCO). Samples a, b, c, and d are spiked with a 0.5um fluorescent bead while e, f, g, and h are spiked with T4 bacteriophage and 0.2, 0.5, and 0.8 um florescent beads (gates C2, C5, and C8 respectively). Sample contents are as follows: a and e contain PBS (blank sample), b and f contain INF (wastewater Influent), c and g contain SEC (secondary treated effluent), while d and h contain EFF (tertiary treated effluent). The total event counts for the depicted sample are located in the lower right-hand corner.

Table S1. Isolated bacteriophage targets and their respective FCM profile ranges (used to validate the placement of the VLP gate)

Virus name	Virus Family	Host	Genome length	Genome type	SSC-H max	FITC-H max	Ref.
T4[1]	Myoviridae	Escherichia coli	~170kbp	dsDNA	700	8500	1
SDS[2]	Siphoviridae	Enterococcus faecalis Yi6-1	~56 kbp	dsRNA	645	2000	2
Ben[2]	Siphoviridae	Enterococcus faecalis Yi6-1	~50 kbp	dsRNA	500	1750	2
KB[3]	Podoviridae	Stenotrophomonas maltiphilia	~43 kbp	dsRNA	525	1600	3
UMP[2]	Podoviridae	Enterococcus faecalis Yi6-1	~20 kbp	dsRNA	625	2800	2
Phi6[4]	Cystoviridae	Pseudomonas syringae	~13 kbp	dsRNA	680	6400	4

References:

(1) ATCC #11303-B4;

(2) Wandro S; Ghatbale P; Attai H; Hendrickson C; Samillano C; Suh J; Dunham SJB; Pride DT; Whiteson K. Phage Cocktails Constrain the Growth of Enterococcus . MSystems 2022, 7.;

(3) Monsibais AN; Tea O; Ghatbale P; Phan J; Lam K; Paulson M; Tran N; Suder DS; Blanc AN; Samillano C; Suh J; Dunham S; Gonen S; Pride D; Whiteson K. Enhanced Suppression of Stenotrophomonas maltophilia by a Three-Phage Cocktail: Genomic Insights and Kinetic Profiling. *BioRxiv* **2024**, 2024.08.14.607921.;

(4) Strain HB104

Table S2. 96-Well Plate setup used for FVM analysis each sampling day

**96-Well Plate FVM Analysis Setup***					
		**PBS**	**EFF**	**SEC**	**INF**
		**1**	**4**	**7**	**10**
**Sample Rep 1**	**A**	PBS Rep 1	EFF Rep 1	SE C Rep 1	INF Rep 1
**POS CTRL T4 10^6 PFU/100mL**	**B**	PBS + POS T4 10^6 PFU/100mL	EFF + POS T4 10^6 PFU/100mL	SEC + POS T4 10^6 PFU/100mL	INF + POS T4 10^6 PFU/100mL
**Sample Rep 2**	**C**	PBS Rep 2	EFF Rep 2	SEC Rep 2	INF Rep 2
**POS CTRL T4 10^7 PFU/100mL**	**D**	PBS + POS T4 10^7 PFU/100mL	EFF + POS T4 10^7 PFU/100mL	SEC + POS T4 10^7 PFU/100mL	INF + POS T4 10^7 PFU/100mL
**Sample Rep 3**	**E**	PBS Rep 3	EFF Rep 3	SEC Rep 3	INF Rep 3
**POS CTRL T4 10^8 PFU/100mL**	**F**	PBS + POS T4 10^8 PFU/100mL	EFF + POS T4 10^8 PFU/100mL	SEC + POS T4 10^8 PFU/100mL	INF + POS T4 10^8 PFU/100mL
**Sample Rep 4**	**G**	PBS Rep 4	EFF Rep 4	SEC Rep 4	INF Rep 4
**Unstained Samples**	**H**	PBS unstained	EFF unstained	SEC unstained	INF unstained

*Two cleaning wells with NovoClean solution (Agilent Technolgies Inc., Santa Clara, CA, USA) and milli-q water were run between each sample well. Cleaning wells (columns 2, 3, 5, 6, 8, 9, 11, and 12) are not depicted in the table.

Table S3. ddPCR viral target oligonucleotide (primer and probe) sequences

Target	ref	Oligonucleotide	Oligonucleotide Sequence (5' -> 3')	ddPCR modification
Pepper Mild Mottle Virus (PMMoV)	1	Forward Primer	GAGTGGTTTGACCTTAACGTTTGA	
		Reverse Primer	TTGTCGGTTGCAATGCAAGT	
		Multiplex Probe	CCTA+C+C+GAAGCA+A+A+TG	5' FAM/ZEN/3'IBFQ (IDT)
crAssphage (CrAss)	2	Forward Primer	CAGAAGTACAAACTCCTAAAAAACGTAGAG	
		Reverse Primer	GATGACCAATAAACAAGCCATTAGC	
		Multiplex Probe	AATAACGATTTACGTGATGTAAC	5'HEX/ZEN/3'IBFQ (IDT)
Tomato Brown Rugose Fruit Virus (ToBRFV)	3	Forward Primer	GGTGGTGTCAGTGTCTGTTT	
		Reverse Primer	GCGTCCTTGGTAGTGATGTT	
		Multiplex Probe	AGAGAATGGAGAGAGCGGACGAGG	5'Cy5/TAO/3'IBRQ (IDT)

"+" symbols represent ligation sites in the nucleic acid sequence

(1) Haramoto E; Kitajima M; Kishida N; Konno Y; Katayama H; Asami M; Akiba M. Occurrence of pepper mild mottle virus in drinking water sources in Japan. *Appl Environ Microbiol* 2013, 79, 7413–7418

(2) Ahmed W; Payyappat S; Cassidy M; Besley C. A duplex PCR assay for the simultaneous quantification of Bacteroides HF183 and crAssphage CPQ_056 marker genes in untreated sewage and stormwater. Environ Int 2019, 126, 252–259.

(3) Caruso AG; Bertacca S; Parrella G; Rizzo R; Davino S; Panno S. Tomato brown rugose fruit virus: A pathogen that is changing the tomato production worldwide. Annals of Applied Biology 2022, 181, 258–274.

Table S4. ddPCR (Bio-Rad QX600) cycling times.

**Cycling step**	Temp (C)	Time	# Cycles
Plate equilibrium	25	3 min	1
Reverse Transcription	50	60 min	1
Enzyme activation	95	10 min	1
Denaturation	94*	30 sec	40***
Annealing/Extension	58	1 min	
Enzyme Deactivation	98	10 min	1
Droplet Stabilization	25	1 min	1

Table S5. ddPCR viral target gBlock (positive control) sequences. gBlocks sourced from Integrated DNA Technolgies INC., Coralville, IA, USA.

Target	gBlock Oligonucleotide sequence
Pepper Mild Mottle Virus (PMMoV)	TTTTCCCGGATGTGTAATACATTAGGCGTAGATCCATTGGTGGCAGCAAAGGTA ATGGTAGCTGTGGTTTCAAATGAGAGTGGTTTGACCTTAACGTTTGAGAGGCCT ACCGAAGCAAATGTCGCACTTGCATTGCAACCGACAATTACATCAAAGGAGG AAGGTTCGTTGAAGATTGTGTCGTCAGACGTAGGTGAGTC
crAssphage (CrAss)	CAGAAGTACAAACTCCTAAAAAACGTAGAGGTAGAGGTATTAATAACGATTTA CGTGATGTAACTCGTAAAAAGTTTGATGAACGTACTGATTGTAATAAAGCTAAT GGCTTGTTTATTGGTCATCTTGAAGATGTTAAAGTTGATTGGGCTACACTGAAA GATGATGTTCAAGGTATGCCTTCATTTGCTGGTATGAGTATTCCTTATC
Tomato Brown Rugose Fruit Virus (ToBRFV)	GGTCTTGTGGTGACAGGTGAATGGAATTTGCCAGATAATTGTCGTGGTGGTGTC AGTGTCTGTTTGGTCGATAAGAGAATGGAGAGAGCGGACGAGGCAACTCTTG CTTCGTACTATACCGCAGCGGCTAAGAAAAGGTTTCAGTTCAAAGTCGTTCCA AATTACAACATCACTACCAAGGACGCAGAAAAGGCAGTTTGGCAAGTACTAG TTAATATTAGAAATGTTAAAATTGC

Table S6. Summary of results across the duration the sampling period for coliphage plaque assays, flow virometry, and ddPCR. FCM data is reported as the median of 4 replicates. ddPCR data is reported as the mean of 3 replicates.

Week	Date	Sample	Somatic coliphage (PFU/ 100mL)	(F+) Specific coliphage (PFU/ 100mL)	Total Event Counts (events/ 100mL)	VLP counts (events/ 100mL)	PMMoV (gc/ 100mL)	CrAss (gc/ 100mL)	ToBRFV(gc/ 100mL)
1	10/05/2023	INF	N/A	N/A	3.92E+10	2.86E+10	4.39E+07	4.45E+07	6.60E+07
1	10/05/2023	SEC	N/A	N/A	4.53E+10	4.38E+10	2.11E+06	ND	2.11E+06
1	10/05/2023	EFF	N/A	N/A	7.27E+10	7.10E+10	2.47E+06	ND	1.26E+07
2	18/05/2023	INF	1.72E+05	2.15E+05	2.50E+10	1.74E+10	4.83E+07	3.48E+07	4.02E+07
2	18/05/2023	SEC	6.00E+02	1.50E+01	3.43E+10	3.29E+10	2.03E+05	ND	2.03E+05
2	18/05/2023	EFF	ND	ND	5.49E+10	5.35E+10	1.91E+04	ND	1.20E+05
3	24/05/2023	INF	1.52E+05	1.97E+05	2.54E+10	1.78E+10	5.88E+07	2.50E+07	1.25E+08
3	24/05/2023	SEC	4.30E+02	N/A	3.06E+10	2.92E+10	3.44E+06	ND	3.44E+06
3	24/05/2023	EFF	ND	ND	6.55E+10	6.43E+10	3.16E+04	ND	2.01E+05
4	31/05/2023	INF	3.44E+05	1.88E+05	1.14E+10	5.46E+09	N/A	N/A	N/A
4	31/05/2023	SEC	2.22E+02	N/A	8.55E+09	7.10E+09	N/A	N/A	N/A
4	31/05/2023	EFF	ND	ND	2.74E+10	2.61E+10	N/A	N/A	N/A
5	07/06/2023	INF	1.42E+05	1.14E+05	2.47E+10	1.66E+10	N/A	ND	5.44E+06
5	07/06/2023	SEC	2.00E+02	1.60E+01	1.71E+10	1.52E+10	1.11E+06	ND	1.11E+06
5	07/06/2023	EFF	ND	ND	4.28E+10	4.11E+10	N/A	N/A	N/A
6	14/06/2023	INF	N/A	N/A	6.81E+10	6.63E+10	3.62E+07	4.72E+07	2.13E+09

6	14/06/2023	SEC	3.30E+02	6.60E+01	4.32E+10	4.14E+10	3.15E+05	ND	3.15E+05
6	14/06/2023	EFF	ND	ND	3.40E+10	2.48E+10	3.11E+04	ND	3.08E+05
7	21/06/2023	INF	4.19E+05	4.44E+04	2.55E+10	1.65E+10	2.70E+07	1.38E+07	1.10E+09
7	21/06/2023	SEC	4.40E+02	2.00E+00	1.98E+10	1.76E+10	9.78E+05	ND	9.78E+05
7	21/06/2023	EFF	ND	ND	4.20E+10	4.01E+10	9.41E+04	ND	9.65E+05
8	29/06/2023	INF	2.85E+05	2.98E+05	1.33E+10	9.22E+09	2.34E+07	1.75E+07	1.12E+09
8	29/06/2023	SEC	6.80E+02	1.10E+01	4.52E+10	4.32E+10	6.89E+05	ND	6.89E+05
8	29/06/2023	EFF	ND	ND	5.77E+10	5.59E+10	7.67E+04	ND	1.22E+06
9	06/07/2023	INF	2.17E+05	1.46E+06	1.64E+10	6.31E+09	1.22E+09	1.03E+07	1.21E+09
9	06/07/2023	SEC	4.71E+02	1.24E+02	6.19E+09	4.95E+09	8.70E+05	ND	8.70E+05
9	06/07/2023	EFF	ND	ND	1.46E+10	1.35E+10	1.51E+04	ND	2.41E+05
10	14/07/2023	INF	1.52E+05	5.12E+05	1.81E+10	1.25E+10	2.54E+07	8.55E+06	1.39E+09
10	14/07/2023	SEC	3.50E+01	1.20E+01	2.38E+10	2.21E+10	6.65E+04	ND	7.51E+04
10	14/07/2023	EFF	ND	ND	4.61E+10	4.47E+10	N/A	N/A	N/A
11	25/07/2023	INF	1.65E+05	3.04E+05	2.09E+10	1.72E+10	1.88E+07	7.53E+07	9.72E+08
11	25/07/2023	SEC	1.80E+02	4.93E+01	1.80E+10	1.69E+10	9.72E+04	ND	1.74E+06
11	25/07/2023	EFF	ND	ND	3.89E+10	3.84E+10	6.28E+03	ND	6.98E+04

Table S7. Coliphage assay results comparison between INF and SEC using Students t-test for somatic and (F+) specific coliphage during 11-week sampling period.

Coliphage type	T-statistic	Mean difference	*p*-value	Significant difference
Somatic	5.87	2.27×10^5	1×10^{-3}	Yes
(F+) Specific	2.31	3.70×10^5	6×10^{-2}	No

Table S8. FVM results of Kruskal-Wallis test and Dunn–Bonferroni post-test for VLP event counts during 11-week sampling period.

**Kruskal-Wallis test results: H-statistic = 33.99, *p* = 4.15 x 10^{-8}**				
**Group 1**	**Group 2**	**Mean difference**	***p*-value**	**Significant difference**
INF	SEC	5.76 x 10^{9}	1.78 x 10^{-1}	No
INF	EFF	2.37 x 10^{10}	3.17 x 10^{-8}	Yes
SEC	EFF	1.79 x 10^{10}	3.78 x 10^{-8}	Yes

Table S9. ddPCR results of Kruskal-Wallis test and Dunn–Bonferroni post-test for PMMoV gene copies during 11-week sampling period.

**Kruskal-Wallis test results: H-statistic = 58.49, *p* = 1.99 x 10^{-19}**				
**Group 1**	**Group 2**	**Mean difference**	***p*-value**	**Significant difference**
INF	SEC	1.65 x 10^{8}	3.00 x 10^{-6}	Yes
INF	EFF	1.65 x 10^{8}	1.58 x 10^{-13}	Yes
SEC	EFF	6.62 x 10^{5}	8.36 x 10^{-3}	Yes

Table S10. ddPCR results of Kruskal-Wallis and Dunn–Bonferroni post-test for ToBRFV gene copies during 11-week sampling period.

**Kruskal-Wallis test results: H-statistic = 53.85, *p* = $2.03x10^{-12}$**				
**Group 1**	**Group 2**	**Mean difference**	***p*-value**	**Significant difference**
INF	SEC	7.69×10^{8}	7.85×10^{-9}	Yes
INF	EFF	7.68×10^{8}	1.50×10^{-10}	Yes
SEC	EFF	8.81×10^{5}	9.00×10^{-1}	No

Table S11. ddPCR results of Kruskal-Wallis and Dunn–Bonferroni post-test for total detected gene copies during 11-week sampling period.

**Kruskal-Wallis test results: H-statistic = 135.19, p = 4.4×10^{-30}**				
**Group 1**	**Group 2**	**Mean difference**	***p*-value**	**Significant difference**
INF	SEC	3.39×10^{8}	1.18×10^{-17}	Yes
INF	EFF	3.39×10^{8}	2.84×10^{-10}	Yes
SEC	EFF	1.11×10^{5}	8.33×10^{-2}	No

S1. Nucleic acid extraction protocol.

After sample collection, nucleic acids were extracted using the MagMAX™ Microbiome Ultra Nucleic Acid Isolation Kit (catalog number #A42357, Thermo Fisher Scientific, Waltham, MA, USA). The ddPCR method used for molecular analysis is as as follows: Samples (4.875 mL) of INF, SEC, and EFF were loaded into a Kingfisher 24-deep well plate and placed in an Eppendorf epMotion liquid handler. Nanotrap® Enhancement Reagent 1 (50 µL, Ceres Nanosciences) was added to the samples followed by incubation for 25 minutes at room temperature. Next, 75 mL of Nanotrap magnetic beads (Nanotrap® Microbiome A Particles, Ceres Nanosciences) were added, and the sample plate was loaded onto a Kingfisher Apex system along with a separate plate containing 400 µL of MagMAX™ Microbiome Lysis Solution. Following the program provided by the manufacturer, samples and magnetic beads were mixed, and the beads were eluted into the lysis solution, collected, and moved to a Kingfisher 96-deep well plate containing a mixture of 500 µL of MagMAX™ Binding Solution and 20 µL of Binding Beads. Using the Kingfisher Apex, the beads are collected and washed in 1 mL MagMAX™ Wash Solution and 1 mL 80% Ethanol before being eluted into 100 µL MagMAX™ Elution Solution in a sterile 1.5 mL polypropylene microcentrifuge tube. The eluted aliquoted was then frozen and -80°C until analysis was completed. Additional information related to the extraction procedures used for ddPCR analysis are reported by Muralidharan et al. (2024).
